# Arterial hypertension in kidney transplantation: huge importance, but few answers

**DOI:** 10.1590/2175-8239-JBN-2022-0109en

**Published:** 2022-10-21

**Authors:** Rafael Naufel de Sá Rebelo, Cibele Isaac Saad Rodrigues

**Affiliations:** 1 Pontifícia Universidade Católica de São Paulo, Faculdade de Ciências Médicas e da Saúde, Programa de Pós-Graduação em Educação nas Profissões da Saúde, Sorocaba, SP, Brazil.

**Keywords:** Kidney Transplantation, Hypertension, Blood Pressure Monitoring, Ambulatory, Goals, Therapeutics, Transplante de Rim, Hipertensão, Monitorização Ambulatorial da Pressão Arterial, Objetivos, Terapêutica

## Abstract

Arterial hypertension (AH) after renal transplantation (RTX) is correlated with worse cardiovascular and renal outcomes, with loss of renal function, decreased graft survival and higher mortality. RTX recipients have discrepant blood pressure (BP) values when measured in the office or by systematic methodologies, such as Ambulatory Blood Pressure Monitoring (ABPM), with significant prevalence of no nocturnal dipping or nocturnal hypertension, white coat hypertension and masked hypertension. The aim of the present study was to review the issue of hypertension in RTX, addressing its multifactorial pathophysiology and demonstrating the importance of ABPM as a tool for monitoring BP in these patients. Treatment is based on lifestyle changes and antihypertensive drugs, with calcium channel blockers considered first-line treatment. The best blood pressure target and treatment with more favorable outcomes in RTX are yet to be determined, through well-conducted scientific studies, that is, in terms of AH in RTX, we currently have more questions to answer than answers to give.

## Resumo

Hipertensão arterial (HA) no póstransplante renal (TXR) se correlaciona com piores desfechos cardiovasculares e renais, com perda de função renal, diminuição da sobrevida do enxerto e maior mortalidade. Receptores de TXR apresentam valores discrepantes de pressão arterial (PA) quando ela é obtida em consultório ou por metodologias sistematizadas, como a Monitorização Ambulatorial da PA (MAPA), com prevalências significantes de ausência de descenso noturno ou hipertensão noturna, hipertensão do avental branco e hipertensão mascarada. O objetivo do presente estudo foi rever a temática da hipertensão no TXR, abordando sua fisiopatologia multifatorial e demonstrando a importância da MAPA como ferramenta de acompanhamento da PA nesses pacientes. O tratamento é baseado em mudanças no estilo de vida e em fármacos anti-hipertensivos, sendo os bloqueadores de canais de cálcio considerados de primeira linha. A melhor meta pressórica e o tratamento com desfechos mais favoráveis no TXR ainda estão por ser determinados, por meio de estudos bem conduzidos cientificamente, ou seja, em termos de HA no TXR temos atualmente mais questões a responder do que respostas a dar.

## Introduction

Arterial Hypertension (AH) is categorized in Brazil as a non-communicable chronic disease, defined by persistent elevation of blood pressure (BP), with Systolic Blood Pressure (SBP) ≥ 140 mmHg and/or Diastolic Blood Pressure (DBP) ≥ 90 mmHg , using the correct technique, on at least in two different occasions without the use of antihypertensive medications^1^. It is classically characterized as the main risk factor for the development of Cardiovascular Diseases (CVD), Chronic Kidney Disease (CKD) and death^2^,^3^.

According to the Kidney Disease Improving Global Outcomes (KDIGO) 2021^4^, arterial hypertension (AH) is considered in renal transplantation (RTX) when the BP is >130/80 mmHg.

The prognostic classification of the renal transplant patient, according to the glomerular filtration rate estimated by formulas such as the Modification Diet in Renal Disease (MDRD) and Chronic Kidney Disease Epidemiology (CKD-EPI), follows the same logic as the CKD patient who includes them in stages 1 to 5, comparing them also according to the degree of albuminuria^4^.

Although RTX corrects many of the pre-transplant changes, AH remains a challenge to be faced in at least 50% of patients, both as an etiology of CKD and as a consequence of the disease, called “de novo hypertension”. This prevalence is quite variable among the different studies in the literature, so that it may be present in up to 70% to 90% of patients who received RTX^5^, ^6^, ^7^, ^8^.

AH after RTX is related to the kidney disease that determined the loss of function of the primitive kidneys and its continuity depends on the disease that affected them. As nephrectomy of primitive kidneys is not usually indicated, they may continue to produce vasopressor substances that maintain the hypertensive process, such as renin, activating the renin-angiotensin-aldosterone system (RAAS)^9^.

Kidney transplant recipients are usually affected by numerous comorbidities of different natures, such as diabetes mellitus (DM), dyslipidemia, obesity, Metabolic Syndrome, among others, in addition to a higher prevalence of adverse events resulting from the use of immunosuppressive drugs, particularly calcineurin inhibitors (CIN) and glucocorticoids, which worsen blood pressure control^10^.

The role of immunosuppressive therapy is vital in preventing rejection and prolonging graft survival, but it can cause AH, DM, dyslipidemia, electrolyte disorders and, paradoxically, nephrotoxicity. Glucocorticoids and CIN can increase sensitivity to vasoconstrictors, activating the RAAS, endothelin and thromboxane A2 (TXA2), in addition to determining sympathetic hyperactivity and sodium retention. On the other hand, they inhibit the synthesis of nitric oxide and prostacyclin, which may increase the formation of free radicals. It has also been reported that CIN can cause thiazide-sensitive sodium chloride cotransporter hyperactivity. Together, these processes cause endothelial dysfunction and contribute to the impairment of organ function^11^, ^12^, ^13^.

Like the CIN, glucocorticoids are important drugs, preventing graft rejection, but they are independent risk factors for the development of AH with a dose-dependent effect, due to the activation of mineralocorticoid receptors, leading to water retention^14^.

A promising line of research is the study of the development of HA post-transplantation when donors are hypertensive and recipients become hypertensive. A systematic review with meta-analysis concluded that this condition increases the risk of post-transplant AH and loss of the transplanted kidney, although more studies are needed to establish this relationship^15^.

The presence of genetic mutations in the Apolipoprotein L1 (APOL1) and Caveolin-1 (CAV-1) genes, present in the donor and in the recipient, is associated with worse outcomes for the renal graft and the development of HA after RTX^16^,^17^.

Another key point is the quality of the transplanted kidney, which can be evaluated through the number of normal and sclerosed glomeruli in the renal biopsy and the calculation of the Kidney Donor Profile Index (KDPI), which uses clinical and laboratory data to classify the donated kidney as normal or expanded criteria kidney, the latter having worse outcomes and greater possibility of AH^18^.

The age factor adds risk to the recipient. It is estimated that for each 10-year increase in donor age, the risk of post-transplant AH increases by 28%^15^,^19^.

After RTX, some factors may also be related to the appearance or worsening of HA in the recipient, such as, for example, the presence of antibody-mediated rejection events, late graft function, and the possibility of partial obstruction of the renal artery at the site of the anastomosis, which implies RAAS hyperactivity, with worsening of the hypertensive process and the need for surgical intervention^20^,^21^.

In addition, the inertia of physicians following these patients is highlighted, a phenomenon recognized in the treatment of primary hypertension, even when BP is outside the pressure target^5^,^7^,^9^,^22^,^23^.

Finally, another relevant issue is the arteriovenous fistula (AVF), the main access route to hemodialysis, which determines a modest but significant decrease in BP, as observed in a meta-analysis of 14 studies. On the other hand, ligation of the AVF after transplantation may determine an increase in BP^24^.

Post-transplant AH is correlated with negative cardiovascular and renal outcomes, with loss of renal function, decreased graft survival and higher mortality from these causes in different countries and continents^10^,^25^,^26^.

**Figure 1:**
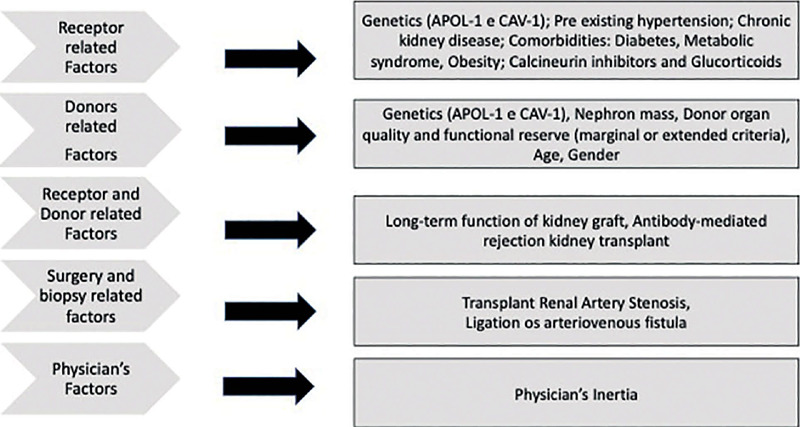
Factors involved in post-transplant arterial hypertension.

Figure 1 summarizes the main pathophysiological aspects involved in RTX AH that depend on numerous factors.

### BP measurement in kidney transplant patients

RTX recipients often present discrepant BP values when it is measured in the office or by systematic methodologies, such as Home Blood Pressure Monitoring (HRBP) or Ambulatory BP Monitoring (ABPM). It is therefore recommended that blood pressure control in kidney transplant recipients be performed using these tools for greater diagnostic and therapeutic safety, considering the AH phenotype found^25^.

The 24-hour MAP allows a rigorous analysis of blood pressure behavior, enabling the detection of attenuation or absence of nocturnal dipping, or even the appearance of hypertension during sleep, common conditions in chronic kidney patients and transplant recipients, which determine greater cardiovascular risk and which can be minimized with the use of chronotherapy, as demonstrated in several studies^27^,^28^.

A satisfactory nocturnal dip is considered to be a drop in SBP and DBP between 10-20% during sleep. When the decrease in BP during sleep remains between 1-10%, it is called attenuated nocturnal dipping; when negative or equal to zero, it is designated nocturnal descent or riser; and when it is present and >20%, it is classified as extreme nocturnal dipping^29^.

Significant prevalence of white coat hypertension (WCH) and masked hypertension (MAH) are frequent in ABPM, which can also occur in the general population and are described as blood pressure variations only observed when measurements are taken outside the office. WCH is characterized when BP is elevated in the office but is normal outside of it. MAH, in contrast, occurs when BP is normal in the office, but high in measurements performed away from this environment^30^.

Sustained AH, also called True Hypertension (TH), occurs when BP is altered in the office and is equally high during HRBP or ABPM. When the BP is within the limits established in the office and in home monitoring (ABPM or HMBP), it is called True Normotension (TN)^30^.

**Figure 2:**
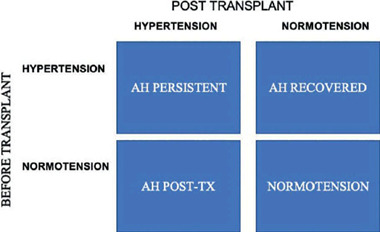
Blood pressure behavior in kidney transplanted patients before and after kidney transplant.

**Figure 3:**
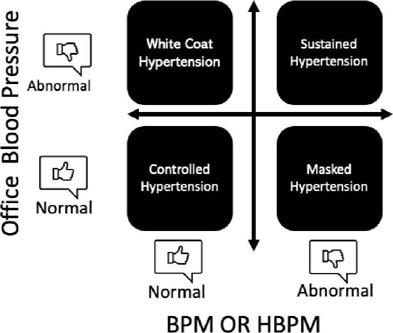
Arterial hypertension phenotype classification, according to ambulatory blood pressure monitoring (ABPM).

Figure 2 demonstrates the different possibilities of blood pressure behavior in kidney transplant recipients, and Figure 3 summarizes the classification of AH phenotypes, according to ABPM findings.

An integrative literature review was carried out using the Newcastle-Ottawa scale to search for clinical studies available in the last 10 years, in English and Portuguese, that portray the theme of arterial hypertension in kidney transplant recipients. Searches were carried out in PubMed, Medline and SciELO electronic databases and search filters were used to include articles published after 2012, with the following descriptors: Renal Transplantation, Arterial Hypertension and Ambulatory Blood Pressure Monitoring. The survey was carried out by two independent researchers in order to answer the question: What are the main ABPM studies in kidney transplant recipients?

This summary can be viewed in Chart 1.

**Chart 1:** Main ABPM studies Performed in kidney transplant recipients in the last 10 years

First author and year of publication	Sample size (n)	Main findings
Korogiannou et al., 2022^31^	136 men and 69 women	Similar office PBP and DBP between genders, but the daytime ABPM was significantly worse in me (prevalence and control). No difference in nighttime B P.
Korogiannou et al., 2021^32^	205	ABPM showed a high prevalence of office HBP (around 90%), MAH and low rates of blood pressor control, in addition to 6.7% of de WCH and 39.5% of MHA (cutoff of 140/90 mmHg) and 5.9% of de WCH and 31.7% of MHA (cutoff of 130/80 mmHg). Low diagnostic BP performance in the office.
Nguyen et al., 2021^33^	57	Daytime ABPM comparable to the standardized measure, automated and alone with AP BP with a 20% incorrect classification rate.
Gluskin et al., 2019^34^	76	*Non dipping* pattern in 73% of the patients.
Mallamaci et al., 2018^25^	260	In 37% of the visits there were disagreements which would cause wrong decision making in 1/3 of the patients.
Kendirlinan et al., 2016^35^	87	MAH in 16.1% and WCH in 24.1%, high rate of patients with mild nighttime dipping (67.8%). LVH in 21.8% of the patients.
Ahmed et al., 2015^36^	98	ABPM discrepancy in 61% of the patients, 58% due to MAH, of which 33% were caused by nocturnal isolated AH, with 42% of conduct change.
Kayrak et al., 2014^37^	11 3	MAH in 39% of the transplanted patients, with a > prevalence among those from deceased donors (40% *vs*. 19%).
Fresnedo et al., 2012^23^	868	36.5% of the recipients had their BP controlled (mean 24 hours < 130/85 mmHg), 65% were considered WCH phenotype.
Ibernon et al., 2012^38^	126	n=65 patients had *non dipping* e pattern and 39 lost the graft.
Wen, 2012^39^	244	Office measures overestimate the values obtained in the 24h and in daytime BP (p < 0.001), which shows the high prevalence of WCH.
Agena et al., 2011^40^	183	Results obtained in the HMBP were closer to those from ABPM than the office measures.

ABPM: ambulatory blood pressure monitoring; MAH: masked arterial hypertension; WCH: white coat arterial hypertension; HMBP: home blood pressure monitoring; LVH: left ventricular hypertrophy.

Korogiannou et al.^32^, in Athens, included 205 stable patients, 136 men and 69 women, who had their blood pressure assessed in the office and by ABPM. The prevalence of AH by office BP was 88.3% or 92.7%, depending on the guideline used (European or American)^31^,^32^, and 94.1% or 98.5% by ABPM. Control rates among hypertensive patients were 69.6% and 43.7% with office BP compared to 38.3% and 21.3% with ABPM, respectively. WCH and MAH were diagnosed in 6.7% and 39.5% of patients at the 140/90 mmHg threshold and in 5.9% and 31.7% of patients at the 130/80 mmHg threshold. Office BP ≥140/90 mmHg had 35.3% sensitivity and 84.9% specificity for the diagnosis of 24-hour BP ≥130/80 mmHg. An office BP ≥130/80 mmHg had 59.7% sensitivity and 73.9% specificity for the diagnosis of 24-h BP ≥125/75 mmHg^33^. In 2022, the same group published data on these patients separated by sex and concluded that men have a higher prevalence and higher BP values in ABPM than women, which may mean a higher risk of cardiovascular and renal outcomes. No significant differences were found in office and nocturnal BP on ABPM^31^.

Nguyen et al.^33^ followed 56 clinically stable transplant patients using BP measurements by two established methods. Daytime ABPM, when compared to automated, standardized and unattended measurement of office BP, showed diagnostic agreement rates of 80%, and can be used as an alternative to ABPM when it is unavailable.

Gluskin et al.^34^ compared in-office and out-of-office measurements by ABPM in transplanted adults. Office BP averaged 128/79 mmHg. During the day, systolic and diastolic BP was 147/85 mmHg and at night, 139/78 mmHg, with a non-dipping pattern in 73% of patients, which was independently and significantly associated with the use of tacrolimus.

The study by Mallamaci et al.^25^ had a longitudinal and cohort design with a follow-up of approximately 4 years. In-office and 24-hour ABPM measurements were obtained. The main findings were that 74% of the patients had nocturnal hypertension (>120/70 mmHg) and the agreement between the office and 24h ABPM, daytime and nighttime BP measurements was unsatisfactory. In 25% of the visits (n=193), the office measurement indicated the need to start or change drug therapy (BP>140/90 mmHg); however, the 24h ABPM was normal (<130/80 mmHg), while that in 94 visits (12%) the 24h ABPM indicated levels compatible with treatment, and the office BP was discrepant. Altogether, in 37% of the visits there were disagreements that would lead to wrong decisions in 1/3 of the patients.

In Turkey, Kendirlinan Demirkol et al.^35^ compared echocardiographic findings with office measurements and daytime ABPM and showed that only 36.8% had agreement between the measurements, with a prevalence of MAH of 16.1% and HAB of 24.1% , in addition to a high proportion of patients with attenuation of nocturnal dipping (67.8%). Left ventricular hypertrophy was found in 21.8% of patients.

Ahmed et al.^36^, through a retrospective study, compared office and residential measurements (ABPM and HMBP) with ABPM disagreement in 61% of patients; 58% of which were due to MAH, of which 33% were due to AH alone at night. Mean systolic BP was 3.6 mmHg and diastolic BP was 7.5 mmHg higher in out-of-office measurements, independently. ABPM results were responsible for behavior change in 42% of cases.

Another Turkish study by Kayrak et al.^37^ showed that mean daytime SBP >135 mmHg and DBP >85 mmHg were the defining values of MAH, which was present in 39% of the patients. Using multivariate analysis, the authors proved that having received RTX from a deceased donor was an independent factor for predicting MAH.

The multicenter RETENAL study conducted in Spain by Fernandez Fresnedo et al.^23^, in 30 transplant centers, included participants with deceased donor kidneys functioning for at least one year, with a mean age of 53.2 years, an estimated GFR ≥ 30 mL/min/1.73 m^2^, serum creatinine < 2.5 mg/dL and follow-up of 5.5 years. Mean office SBP and DBP were 140.2 and 80.4 mmHg, respectively; and 66% of office measurements were ≥ 130/80 mmHg. The mean 24-hour ABPM was 131.5 and 77.4 mmHg for SBP and DBP, respectively. According to ABPM, only 36.5% of the recipients had controlled BP (mean 24 hours < 130/85 mmHg), 65% were considered to have a WCH phenotype. Considering the results, the authors recommend ABPM for medication adjustment and adequate BP control.

Ibernon et al.^38^ found that reversal of nocturnal descent was associated with inflammation and graft loss in 39 recipients with this phenotype and more than half of the patients (n=65) exhibited a non-dipping pattern.

Wen and Gourishankar^39^ found a mean SBP in the office of 137.1 mmHg and DBP of 79.9 mmHg. At ABPM, these values were respectively 131.3 mmHg and 75.37 mmHg; being 133.5 mmHg and 77.4 mmHg the daytime average. Thus, the office measurements overestimate the values obtained in the 24 hours and in the daytime BP (p < 0.001), which demonstrates the high prevalence of WCH.

National data published in 2011 by Agena et al.^40^ analyzed RTX with kidneys from living (46%) or deceased (54%) donors, with a mean age of 50 years; 54% of men and transplant time of 57 months. Using office BP, 56.3% had uncontrolled BP, with a mean of 138.9/82.3 mmHg, while using HMBP this percentage decreased to 44.8%, with a mean of 131.1/78.5 mmHg. In ABPM, only 36.1% had control with a mean of 128.8/80.5 mmHg. The authors concluded that the results obtained by HMPB were closer to ABPM than those obtained in the office, being recommended to detect uncontrolled BP. The use of ABPM, enabling greater diagnostic accuracy, can reduce the exaggerated prescription of drugs that cause hypotension and low kidney blood flow, greater possibility of drug interactions and poor treatment compliance. On the other hand, the finding of non-dippers, not identifiable by office measurements, facilitates the proper management of these cases and the prevention of graft loss, the onset of cardiovascular comorbidities and reduced mortality^6^,^41^.

A point to be stressed is the high complexity of the ABPM analytical report and the dependence of a qualified physician to report the exam, which is susceptible to interpretive errors^29^.

In conclusion, although with different prevalence, it is correct to say that renal transplant patients should always be followed up with regular ABPM, which is an international recommendation. Phenotypes other than what is considered normal are prevalent, and these patterns may be correlated with worse cardiovascular and graft outcomes, possibly also with mortality, as in non-transplanted hypertensive adults, for reasons that are still unknown^41^.

This statement is also supported by a systematic review with meta-analysis by Pisano et al., which included 22 studies (2,078 participants). Among 12 studies that evaluated data on renal outcomes, ten indicated that BP assessed by ABPM was a better predictor of decline in renal function than office measurements. Twelve studies analyzed the relationship between different BP records and target organ damage, and found robust correlations between echocardiographic abnormalities and vascular damage markers with 24h ABPM but not with office BP. In addition, the abnormal circadian BP pattern (non-dippers and reverse dippers) identified RTX receptors at risk for loss of renal function and cardiovascular abnormalities^42^.

The phenotypes diagnosed by ABPM are associated with more adequate decision-making and with outcomes, such as target organ damage and adverse renal events, translated into worsening proteinuria, progressive decrease in glomerular filtration rate and cardiovascular compromise.

Studies that assess AH in RTX contribute to a better understanding of this frequent situation in transplant patients, and approaches aimed at controlling the different phenotypes have the potential to minimize damage.

### Treatment goal

In the absence of specific randomized and controlled studies in this population, comparing different long-term treatments in relation to renal and cardiovascular outcomes, we should use existing blood pressure targets for uncontrolled hypertensives^1^,^43^.

It is interesting to note that the ESC/ESH – European Society of Cardiology and European Society of Hypertension (2018)^44^, the ISH – International Society of Hypertension (2018)^45^, and the DBHA: Brazilian Guidelines on Hypertension (2020)^1^ did not set goals in its guidelines, showing this knowledge gap. Only the KDIGO – Kidney Disease Improving Global Outcomes and the consensus position of the ESH – Hypertension in Kidney Transplantation, developed by the working group on hypertension and the kidney, both from 2021, recommend that blood pressure levels in these patients be kept < 130/80 mmHg^4^.

### Non-drug treatment of arterial hypertension in renal transplant recipients

Lifestyle changes (LSC) are always the first measures known to be effective in the treatment of AH in RTX, which do not differ from those recommended for AH in the general population^4^.

Ideally, every renal transplant patient should be treated by a multidisciplinary team, which is not always the reality of the services that carry out this follow-up. Thus, it is extremely important for the patient to be part of the process, as their lifestyle can directly affect hard outcomes, such as RTX function and mortality^46^.

Briefly, LSC include a healthy diet, rich in fruits and vegetables, low in saturated fat and cholesterol, with grains, low in protein and low in sodium; regular physical activity, according to cardiovascular and respiratory capacity, preferably 150 minutes/week; maintenance of body mass index < 25 kg/ m^2^; restriction of alcohol consumption and smoking cessation; in addition to guidance on medications that should be avoided^4^,^10^,^47^,^48^.

In recent years, several dietary trends have emerged aimed at reducing cardiovascular risk, anti-inflammatory effects and weight loss. Among them, plant-based diets, intermittent fasting, low-carbohydrate/keto diet and juices of different compositions have received media attention, but care and knowledge about the potential risks and benefits in kidney transplant recipients are needed^49^.

A non-pharmacological measure that is widely encouraged and universally recommended for hypertensive patients is the use of a low-sodium diet, a change that is easy to implement, effective and cost-free. The 2021 KDIGO document suggests that sodium intake should be < 2 g of sodium per day (or < 5 g of salt per day) in patients with AH and CKD, with no specific recommendation for transplant recipients^4^.

It is noteworthy that studies in the general population, but also in kidney transplant recipients, have associated the Mediterranean Diet with favorable clinical outcomes^50^,^51^.

Regarding the DASH diet, in a prospective cohort study with 632 clinically stable renal transplant recipients, greater compliance to the DASH diet was associated with a lower risk of renal function decline (hazard ratio 0.95; P = 0.008), as well as lower mortality for all causes (hazard ratio 0.95; P = 0.01)^52^. A recent review details the main positive and negative aspects of each type of diet^49^.

Physical exercise seems to be safe in RTX recipients and itis associated with a better quality of life and exercise capacity^53^. However, the available data are characterized by great variability in patient characteristics; interventions performed and outcome measures investigated in the studies, most of them through questionnaires^54^; as well as the use of small cohorts, but which is very similar to the other studies found. These limitations suggest the need for more research in this area, with an adequate sample size, using objective and standardized measures of physical activity and fitness in RTX recipients and the time required for them to produce beneficial blood pressure effects and improve outcomes^55^.

### Drug treatment of arterial hypertension in renal transplant recipients

Pharmacological treatment should be individualized and include antihypertensive drugs of different pharmacological classes until the recommended goals are reached. Risk-modifying variables such as sociodemographic profile, presence of comorbidities, stage of CKD and presence of albuminuria should always be compared. In addition, dosage convenience, availability, greater efficacy and tolerability, and fewer adverse effects are considered. Often, AH will be characterized as resistant or refractory, requiring the association of drugs with synergistic effects, which may be an option from the beginning, including in association^4^,^56^,^57^.

KDIGO 2021, as well as other authors, including a systematic review with meta-analysis and the 2021 European Society of Hypertension consensus, indicate that the first-line drugs are calcium channel blockers (CCB), as they improve graft function and reduce its loss. Its nocturnal use has shown good performance in reversing the altered sleep pattern. They are indicated especially in the first few months of RTX when the risk of acute kidney injury is greater, and RAAS blockers, particularly angiotensin II AT1 receptor blockers (ARBs), when renal function is stable, because these drug classes reduce graft loss when compared to the others. However, they make it clear that there is a lack of studies that support an evidence-based recommendation, since kidney transplant recipients are very often excluded from randomized clinical trials comparing outcomes^4^,^13^,^56^,^58^, ^59^, ^60^, ^61^.

Transplant patients with coronary heart disease and/or arrhythmias may benefit from beta-blockers or alpha and beta-blockers such as carvedilol. In benign prostatic hyperplasia, alpha-blockers are well indicated. In all cases in which hypervolemia or edematous states occur, thiazide or loop diuretics can be associated, including mineralocorticoid receptor antagonists, always with monitoring of serum potassium levels and kidney function^13^,^48^,^56^.

SGLT2 inhibitors and selective mineralocorticoid receptor antagonists are promising for use in RTX, as they cause nephroprotection, regardless of antihypertensive action, and can potentially be beneficial, but this is still a hypothesis to be unveiled, opening the way for studies with that population.

**Figure 4:**
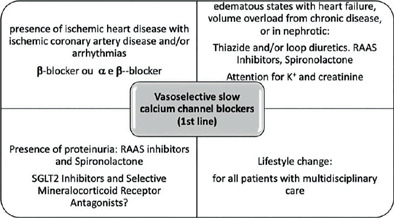
Main aspects of hypertension treatment in kidney transplanted patients.

The main aspects of treatment are summarized in Figure 4 and it is important to highlight that individualized treatment is always the best option.

## Final remarks

Transplantation represents an alternative treatment for CKD, improving the quality and quantity of life and reducing the risk of cardiovascular complications, outcomes related to chronic diseases, in addition to avoiding adverse events related to the dialysis procedure itself. In addition, it allows the reintegration of the individual into social life, increases the possibility of returning to work practices, in addition to improving physical and mental condition^62^.

Thus, like other chronic patients, transplant patients require close medical monitoring and a multidisciplinary team, compliance to an individualized care plan and to LSC, with constant checking of associated and controllable factors, such as hypertension and DM, both of which are highly prevalent in these individuals, as cause or consequence^63^.

In this sense, it is worth mentioning the complexity of compliance to treatment of an individual with a chronic disease in their self-care^64^. Considered a multifactorial process, lack of compliance to RTX has been documented as the second most prevalent cause of chronic graft loss, and this aspect does not differ regarding the treatment of AH^65^.

The correct diagnosis of AH by ABPM is able to unmask phenotypes that are impossible to determine without this resource, providing better blood pressure control. Although we still have many questions about the best way of diagnosing AH in kidney transplant recipients, the data in the literature enable us to advocate emphatically in favor of its use, or alternatively, HMBP when ABPM is unavailable, for diagnosis and follow-up^33^,^36^,^45^,^66^,^67^.

Technologies such as renal sympathetic denervation and baroreceptor stimulation, not discussed here, should not be used routinely, but can be considered in cases of resistant or refractory AH.

Finally, we conclude that the best blood pressure target and treatment with more favorable outcomes in RTX are yet to be determined, that is, we currently have more questions to answer than answers to give in this complex tangle.
